# Advances in functional nanomaterials and piezoelectric biomaterials for personalized intramedullary fixation: addressing age-related orthopedic challenges

**DOI:** 10.3389/fchem.2026.1909527

**Published:** 2026-09-01

**Authors:** Fei Wang, Fu-Ming Wang, An-Yun Guo, Li Wang, Jian Shang

**Affiliations:** 1 Shenzhen University General Hospital, Shenzhen, Guangdong, China; 2 Hospital of Southern University of Science and Technology, Shenzhen, Guangdong, China

**Keywords:** age-related orthopedic challenges, femoral isthmus, functional nanomaterials, personalized intramedullary fixation, piezoelectric biomaterials

## Abstract

Femoral fractures represent a major global orthopedic burden, particularly among elderly and osteoporotic populations, and are associated with substantial morbidity, mortality, and healthcare costs. Intramedullary fixation, in which a metallic nail is inserted into the medullary canal to stabilize fractured bone, remains the clinical gold standard because it provides load-sharing stabilization through a minimally invasive approach. Nevertheless, implant–bone mismatch, cortical impingement, fixation instability, infection, and delayed osseointegration continue to compromise long-term outcomes owing to patient-specific anatomical variability, age-related skeletal remodeling, and the limited biological activity of conventional implants. This review provides a comprehensive overview of femoral isthmus morphology, age-dependent anatomical remodeling, and their implications for personalized intramedullary fixation. Particular emphasis is placed on recent advances in functional biomaterials designed to improve implant performance and bone regeneration. Representative strategies include bioactive ceramic coatings (e.g., barium titanate and hydroxyapatite), piezoelectric ceramics, electroactive polymers such as poly (vinylidene fluoride) and poly (L-lactic acid), nanostructured coatings, antibacterial interfaces, multifunctional composite scaffolds, and ultrasound-responsive platforms. These materials have demonstrated the ability to regulate osteoblast proliferation and differentiation, enhance osseointegration, modulate inflammatory responses, inhibit bacterial colonization, and accelerate bone healing by providing biochemical, topographical, and electromechanical stimulation at the bone–implant interface. Furthermore, this review discusses emerging technologies, including additive manufacturing, artificial intelligence-assisted implant design, digital twin modeling, shape-adaptive materials, and patient-specific fixation strategies, which collectively offer new opportunities for precision orthopedic care. By integrating advances in femoral anatomy, biomechanics, materials chemistry, nanotechnology, and piezoelectric bioengineering, this review highlights the development of intelligent and multifunctional intramedullary fixation systems that improve implant integration, promote bone regeneration, and address the growing clinical demands of an aging population.

## 1 Introduction

Femoral fractures represent one of the most significant orthopedic injuries worldwide, imposing a substantial clinical, socioeconomic, and public health burden. The global incidence of hip and femoral fractures continues to rise owing to population aging and the increasing prevalence of osteoporosis, with more than 1.6 million hip fractures occurring annually worldwide, a figure projected to exceed 6 million cases by 2050. These injuries are associated with high morbidity, mortality, prolonged hospitalization, loss of independence, and healthcare expenditures exceeding billions of dollars each year, particularly among elderly populations. Consequently, optimizing fracture fixation and improving long-term functional outcomes have become major priorities in contemporary orthopedic practice (Park et al., 2024; Wu et al., 2021). Intramedullary nailing remains the gold-standard treatment for most femoral shaft fractures because of its minimally invasive approach, favorable load-sharing biomechanics, and consistently high union rates (Rosa et al., 2019). Nevertheless, complications including implant-bone mismatch, cortical impingement, malalignment, fixation instability, and delayed healing continue to compromise clinical outcomes, particularly in elderly and osteoporotic patients (Dong et al., 2024). Increasing evidence suggests that these complications arise not only from surgical or material-related factors but also from the inability of standardized implant designs to adequately accommodate the complex three-dimensional anatomical variability of the femoral canal (Rosso et al., 2025).

A key factor governing the success of intramedullary fixation lies in the complex geometry of the femur, particularly the medullary canal and femoral isthmus, which represent critical regions for implant anchorage and load transfer (Cui et al., 2025). Traditional implant design has largely assumed simplified cylindrical or circular canal geometry, overlooking the true 3D variability of femoral morphology. Recent advances in high-resolution computed tomography and 3D reconstruction have revealed that the femoral isthmus exhibits a more complex elliptical configuration, with distinct long and short axis dimensions that vary across individuals and across the lifespan (Ma et al., 2026). This anatomical complexity plays a decisive role in determining implant fit, stability, and stress distribution at the bone-implant interface. Conventionally, intramedullary nails are fabricated from metallic biomaterials such as 316L stainless steel, titanium and titanium alloys (Ti-6Al-4V), and cobalt-chromium (Co-Cr) alloys because of their excellent mechanical strength, corrosion resistance, fatigue durability, wear resistance, and long-term clinical reliability (Makuochukwu and Ilemona, 2026; Wang et al., 2024). Despite these advantages, their high elastic modulus relative to cortical bone often results in stress shielding, impaired physiological load transfer, insufficient osseointegration, and implant-related complications. Furthermore, after implantation, these metallic devices are exposed to a complex physiological microenvironment comprising body fluids, inflammatory mediators, immune cells, fluctuating pH, oxidative stress, and continuous mechanical loading, where conventional materials largely function as passive structural supports without actively regulating biological responses or adapting to patient-specific anatomical variations (Ghiban et al., 2016). To overcome these limitations, increasing attention has been directed toward next-generation orthopedic biomaterials, including hydroxyapatite (Ca_10_(PO_4_)_6_(OH)_2_) and bioactive glass coatings, antibacterial metallic nanoparticles (Ag, Cu, Zn), biodegradable polymers such as poly (L-lactic acid) (PLLA) and polycaprolactone (PCL), electroactive polymers including poly (vinylidene fluoride) (PVDF), piezoelectric ceramics such as barium titanate (BaTiO_3_), and multifunctional nanocomposite coatings (Chen and Wang, 2026; Liang et al., 2024). These advanced materials provide bioactivity, antibacterial performance, osteoinductive capability, controlled therapeutic delivery, and electromechanical stimulation that collectively enhance osseointegration and bone regeneration (Deng et al., 2025). Such functional advantages become increasingly important with aging, as the femur undergoes continuous structural remodeling characterized by cortical thinning, endosteal expansion, alterations in femoral curvature, and deterioration of bone quality, further increasing the risk of implant-bone mismatch and fixation failure.

In addition to geometric considerations, the femur undergoes continuous structural remodeling with aging, influenced by cortical thinning, endosteal expansion, and changes in overall femoral curvature (Chen Y. et al., 2026). These age-related transformations not only alter the spatial position of the femoral isthmus along the shaft but also modify its cross-sectional geometry, gradually shifting from a more elliptical toward a relatively circular profile in older individuals. Such dynamic anatomical evolution introduces significant challenges for fixed geometry implants, particularly in elderly and osteoporotic patients where bone quality is already compromised. As a result, the mismatch between implant design and patient anatomy becomes a major underlying contributor to fixation instability and postoperative complications (Zhou et al., 2024).

To overcome the limitations associated with conventional intramedullary fixation systems, recent advances in materials chemistry and nanotechnology have introduced new opportunities to transform orthopedic implants from passive mechanical supports into biologically interactive therapeutic platforms. Functional surface modifications, bioactive ceramic coatings, antibacterial nanostructures, osteogenic interfaces, and stimulus-responsive materials have demonstrated considerable potential to enhance implant–bone interactions, improve osseointegration, reduce infection-related complications, and promote bone regeneration (Hu et al., 2023; Lin et al., 2025; Xu C. et al., 2025; Yang X. et al., 2024). These emerging strategies provide a pathway toward addressing not only mechanical challenges associated with implant fixation but also the biological limitations of aging and compromised skeletal environments.

**SCHEME 1:**
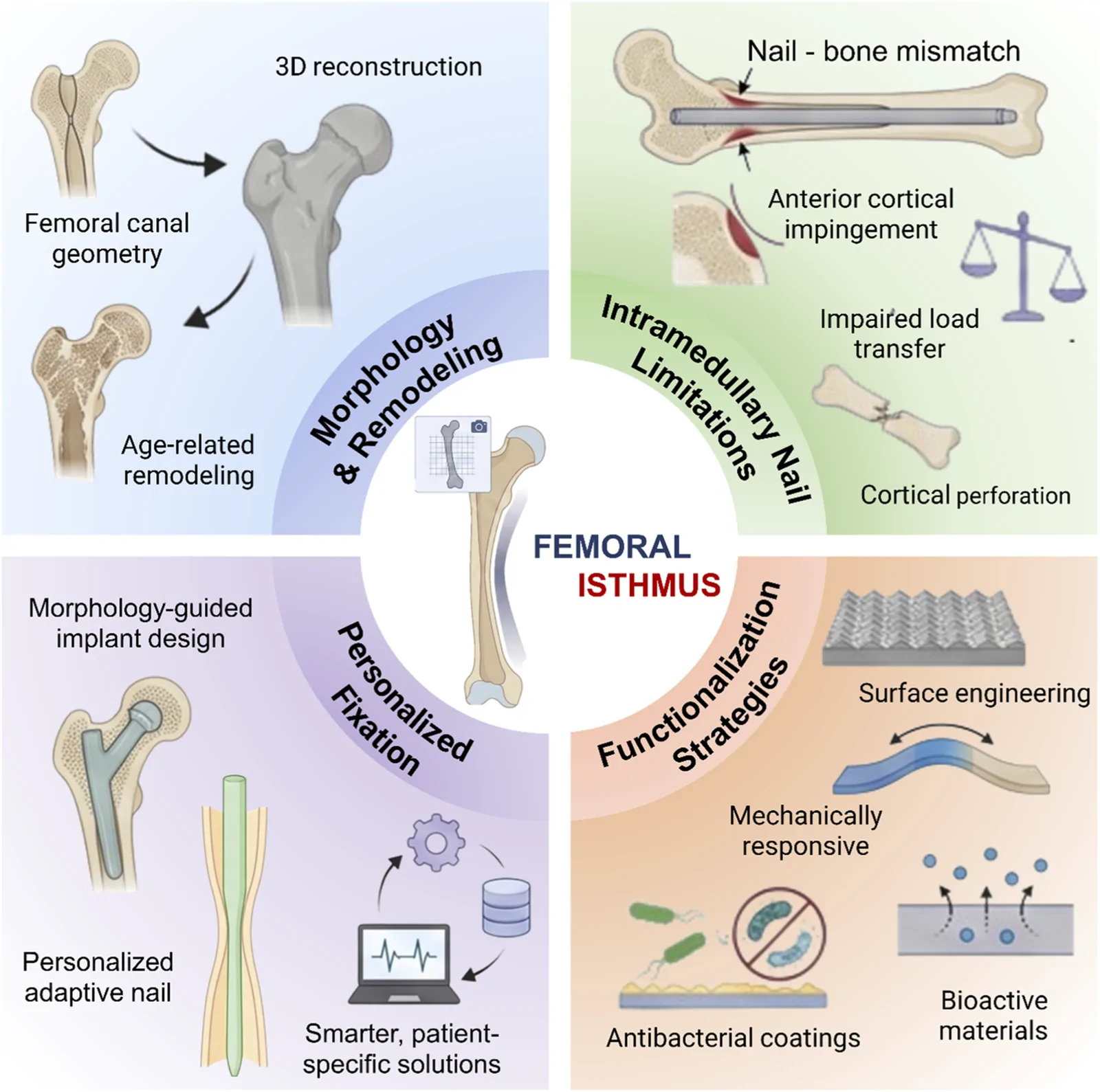
The schematic illustration of the review that summarizes the key themes discussed in this review, including femoral isthmus morphology and aging, limitations of conventional intramedullary nails, functional material strategies, enabling technologies, and future perspectives toward personalized fixation.

In this review, we provide a comprehensive and critical discussion of the interconnected anatomical, biomechanical, and materials-related factors governing the performance of femoral intramedullary fixation systems. Rather than simply summarizing recent advances, this work examines how femoral anatomical variability, age-associated skeletal remodeling, implant design constraints, and bone-implant interactions collectively contribute to clinical challenges, including implant-bone mismatch, cortical impingement, stress concentration, fixation instability, delayed healing, and implant failure. Furthermore, we discuss the evolution of conventional metallic implants and highlight how emerging functional nanomaterials, bioactive coatings, and intelligent implant technologies are reshaping the future of orthopedic fixation. Particular attention is given to piezoelectric biomaterials as a promising class of functional orthopedic materials capable of converting mechanical stimuli generated during physiological activities into localized electrical signals that mimic the natural electromechanical environment of bone. Unlike traditional implant materials that primarily provide structural support, piezoelectric platforms offer active biological regulation by promoting osteogenic differentiation, enhancing mineralization, improving osseointegration, modulating inflammatory responses, and accelerating tissue regeneration. Recent developments in piezoelectric ceramics, polymers, nanofibrous materials, composite systems, and ultrasound-responsive electroactive platforms have further expanded their potential for next-generation orthopedic applications, particularly in elderly populations with reduced regenerative capacity. By integrating anatomical knowledge, orthopedic biomechanics, advanced functional nanomaterials, and piezoelectric bioengineering, this review establishes a multidisciplinary framework for advancing intelligent orthopedic implants. Ultimately, the convergence of precision anatomy and multifunctional material design may enable the transition from conventional fixation devices toward adaptive regenerative platforms capable of improving implant compatibility, accelerating bone healing, reducing complications, and delivering personalized therapeutic outcomes for diverse patient populations (Scheme 1).

### 1.1 Literature selection strategy

This article was developed as a narrative review to provide a comprehensive and critical overview of recent advances in personalized intramedullary fixation, functional nanomaterials, and piezoelectric biomaterials for orthopedic applications. Relevant literature was identified through searches of major scientific databases, including PubMed, Web of Science, and Scopus. Publications from approximately the past decade were primarily considered, while earlier landmark studies were included when necessary to explain fundamental concepts, material evolution, and clinical development. Search terms included combinations of “femoral fracture,” “intramedullary nail,” “femoral isthmus,” “implant-bone mismatch,” “orthopedic biomaterials,” “nanostructured coatings,” “bioactive materials,” “piezoelectric biomaterials,” and “stimulus-responsive implants.” Selected studies were evaluated based on their relevance to anatomical characterization, implant biomechanics, material design, biological responses, and emerging strategies for next-generation orthopedic fixation. This approach enabled integration of multidisciplinary evidence while maintaining the conceptual framework of a narrative review.

## 2 Morphological characteristics of the femoral isthmus and their clinical significance

The femoral isthmus represents the narrowest region of the medullary canal and serves as a critical anatomical landmark for intramedullary fixation. Because this region often provides the primary mechanical interface between the implant and surrounding bone, its morphology strongly influences implant fit, load transfer, rotational stability, and overall fixation performance (Yang et al., 2021). Despite its clinical importance, conventional intramedullary nail designs have historically been developed based on simplified geometric assumptions that do not fully capture the anatomical complexity and variability of the femoral canal. Recent advances in computed tomography, three-dimensional imaging, and anatomical modeling have substantially improved understanding of femoral canal morphology (Chethan, 2018). These investigations have demonstrated that the geometry of the femoral isthmus exhibits considerable interindividual variability and is influenced by factors such as sex, age, skeletal size, ethnicity, and overall femoral morphology. Moreover, increasing evidence suggests that the cross-sectional shape of the isthmus cannot always be adequately represented by a simple circular geometry, highlighting the limitations of standardized implant designs intended to accommodate a broad patient population.

The morphology of the femoral isthmus is also closely associated with other anatomical characteristics of the femur, including femoral length, canal dimensions, cortical thickness, and anterior bowing (Zhang R. y. et al., 2021). These relationships are clinically important because they influence implant insertion, nail alignment, stress distribution, and the risk of complications such as cortical impingement, malalignment, and fixation instability. Furthermore, age-related skeletal remodeling and osteoporosis can progressively alter femoral geometry, creating additional challenges for achieving optimal implant-bone conformity in elderly patients (Fontenla et al., 2026). A comprehensive understanding of femoral isthmus morphology is therefore essential for improving implant design and surgical planning. Recognition of anatomical variability provides an important foundation for the development of patient-specific fixation strategies, anatomically adaptive implants, and next-generation orthopedic systems that better accommodate the diverse morphological characteristics encountered in clinical practice.

### 2.1 Anatomical architecture and biomechanical function of the femoral isthmus

During intramedullary fixation procedures, the femoral isthmus, the narrowest part of the femoral medullary canal, is a crucial anatomical landmark (Rivas et al., 2025). The isthmus serves as the primary contact zone between the intramedullary nail and the surrounding cortical bone due to its smaller canal diameter, which significantly improves implant stability, load transfer, and rotational control (Shi et al., 2025). Adequate fixation is typically thought to depend on the intramedullary nail successfully engaging the isthmus, especially in cases of long bone fractures where distal stabilization is necessary. Beyond its significance in surgery, the femoral isthmus is essential to the biomechanical distribution of physiological loads along the femoral shaft. The cortical shell and medullary structure transfer the stresses produced within the femur during activities like walking, climbing stairs, and bearing weight (Xu W. et al., 2025). Stress distribution patterns, implant-bone interactions, and overall mechanical performance after fracture fixation are all influenced by geometric features of the isthmus. Therefore, accurate isthmus morphology characterization is crucial for improving implant design and reducing surgical problems.

In the past, the femoral isthmus has frequently been defined as a medullary canal narrowing that is roughly round. The design philosophy of the majority of commercially available intramedullary nails, which typically have circular cross-sectional geometries, has been influenced by this simplified description (Heinecke et al., 2025). However, mounting data from sophisticated imaging and 3D reconstruction methods indicate that the femoral isthmus’s shape is far more complicated than previously thought. Implant fit and fixation results may be greatly impacted by variations in canal form, diameter, curvature, and geographic position (Li et al., 2025).

As orthopedic surgery shifts toward individualized treatment approaches, it is becoming more and more crucial to comprehend these anatomical features. In addition to interindividual variability, age-related changes in bone morphology that may modify the mechanical environment of fracture repair must be taken into account by modern implant systems. To create next-generation intramedullary fixation methods, a thorough assessment of the femoral isthmus architecture is therefore essential.

### 2.2 3D reconstruction techniques for quantitative evaluation of femoral morphology

Advances in CT and digital reconstruction technologies have renovated the study of femoral anatomy (Liang and Liew, 2025). Projection errors, picture magnification, and the inability to precisely depict the three-dimensional complexity of skeletal structures are common limitations of outdated radiography measurements. On the other hand, accurate viewing and quantitative evaluation of both cortical and cancellous bone architecture are made possible by high-resolution CT in conjunction with specialized reconstruction software (da Silva et al., 2025). Recent research has employed 3D reconstruction techniques to evaluate a wide range of femoral parameters, including femoral length, neck shaft angle, anteversion angle, cortical thickness distribution, and anterior bow curvature. These methods provide highly reproducible measurements and enable detailed analysis of anatomical characteristics that are difficult to quantify using typical imaging modalities. Furthermore, digital reconstruction enables virtual sectioning of the femur in any desired position, making it particularly useful for describing the femoral isthmus.

**FIGURE 1:**
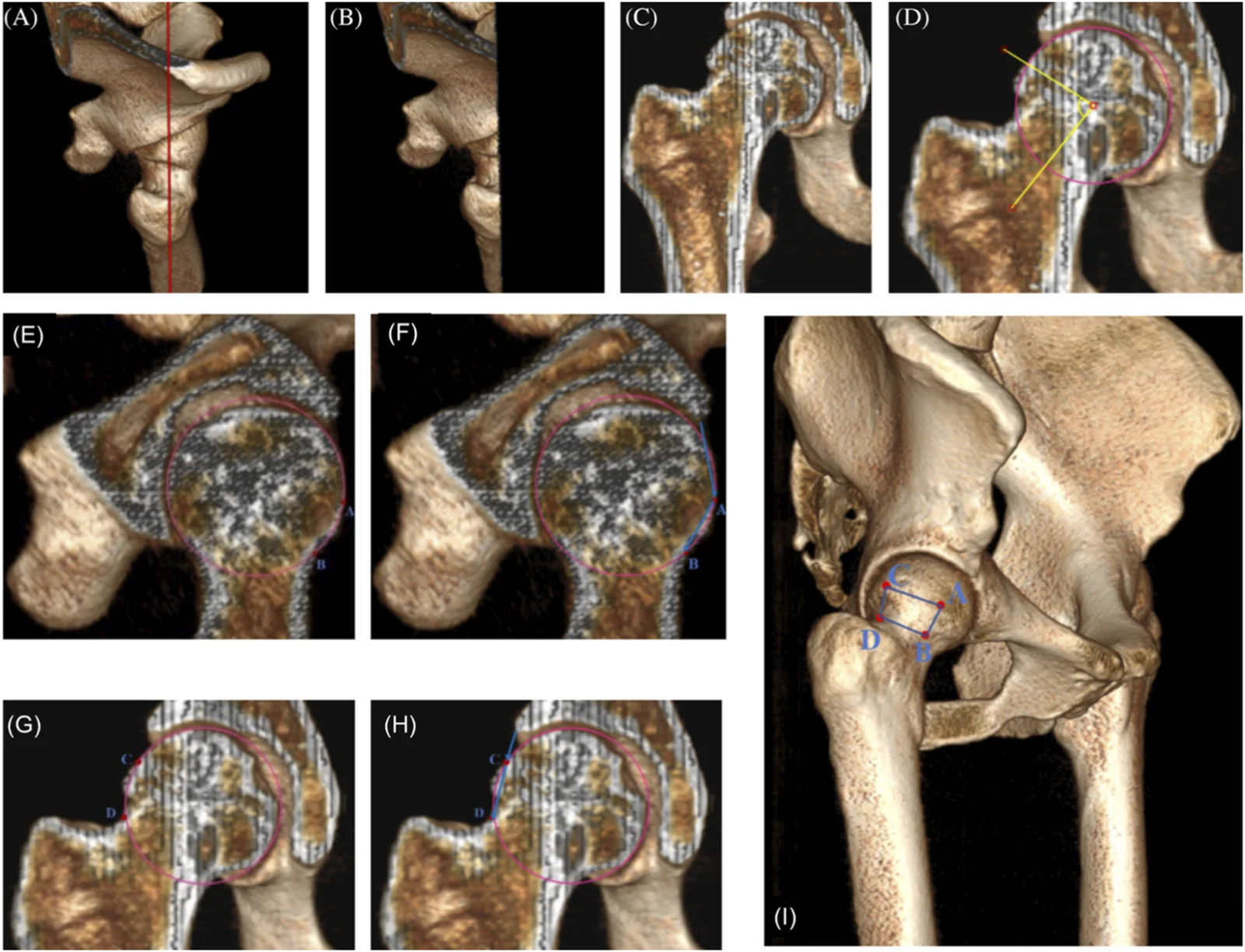
**(A‐D)** Representative workflow for three-dimensional computed tomography‐based quantitative evaluation of femoral morphology and preoperative planning. **(E‐I)** The figure illustrates three‐dimensional reconstruction of the proximal femur, measurement of the lateral α angle, identification of anatomical landmarks, and quantitative delineation of the target femoroplasty region for cam‐type femoroacetabular impingement syndrome. These reconstruction techniques demonstrate the capability of three‐dimensional imaging to characterize femoral geometry and support patient‐specific orthopedic planning accurately. Adapted with permission from Orthopaedic Surgery. Copyright ©, Wiley (Zheng et al., 2025).

Recent advances in imaging and anatomical characterization have revealed that the femoral canal is a complex and highly variable structure influenced by factors such as femoral length, curvature, cortical thickness, and isthmus morphology (Zhao X. et al., 2026). Accumulating evidence suggests that simplified geometric assumptions used in conventional implant design do not adequately reflect this anatomical diversity, which may contribute to implant-bone mismatch and fixation-related complications. Consequently, a more comprehensive understanding of femoral morphology has become increasingly important for the development of personalized and anatomically adaptive intramedullary fixation strategies (Zhu et al., 2026). As illustrated in Figure 1, modern three-dimensional computed tomography-based reconstruction enables accurate visualization of femoral anatomy, quantitative morphometric measurements, and patient-specific preoperative planning, thereby providing a robust anatomical foundation for precision orthopedic interventions (Zheng et al., 2025).

### 2.3 Age-related remodeling of the femoral canal and isthmus

**FIGURE 2:**
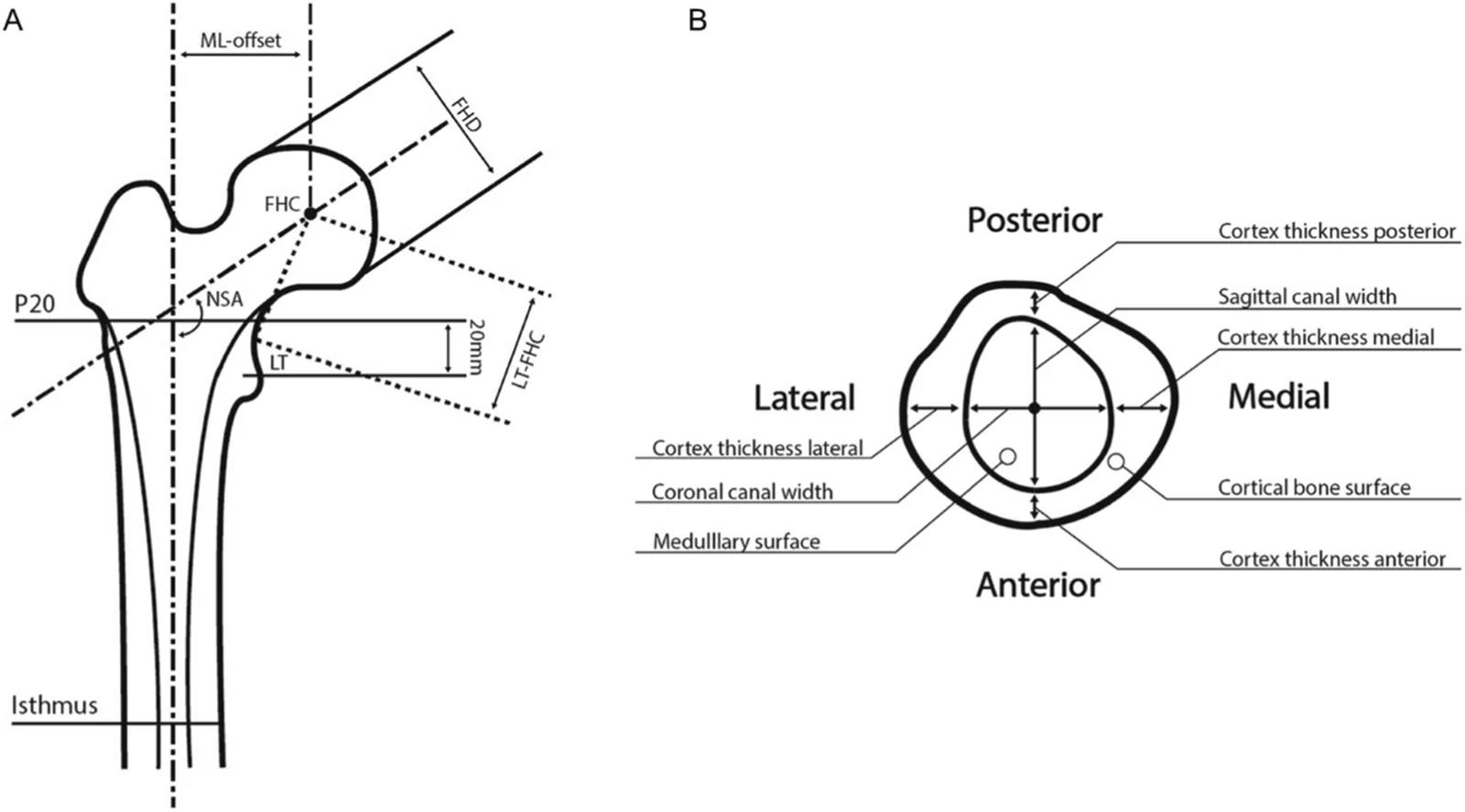
Schematic illustration of the morphometric parameters evaluated in the proximal femur. **(A)** External anatomical parameters and the cross-sectional locations used for internal geometric measurements. **(B)** Internal morphometric parameters assessed at each cross-sectional level. FHC, femoral head center; LT, lesser trochanter; FHD, femoral head diameter; NSA, neck–shaft angle; ML-offset, mediolateral offset; LT-FHC, distance between the lesser trochanter and the femoral head center; P20, cross-sectional level located 20 mm proximal to the lesser trochanter; Isthmus, cross-sectional level corresponding to the femoral isthmus. Adapted with permission from Journal of Orthopaedics. Copyright ©, Elsevier (Veldman et al., 2025).

Age-related structural remodeling of the femoral canal plays a critical role in determining the mechanical environment and long-term performance of intramedullary fixation systems (Xu M. et al., 2026). Recent advances in three-dimensional computed tomography-based morphometric analysis have substantially improved the understanding of proximal femoral anatomy and its implications for orthopedic implant design (Abdul-Hafez et al., 2026). Multiple studies have demonstrated that three-dimensional imaging enables accurate characterization of the femoral canal, cortical architecture, and isthmus morphology, providing anatomical information that cannot be reliably obtained from conventional radiographs. Such approaches have become increasingly important for evaluating patient-specific anatomy, optimizing implant selection, and reducing implant-bone mismatch during intramedullary fixation (Welling et al., 2026). A representative example is illustrated in Figure 2, adapted from Veldman et al., who employed three-dimensional CT reconstructions to quantify both external proximal femoral anatomy and internal canal morphology in very elderly individuals. Their analysis demonstrated that commonly used external anatomical parameters, including the neck-shaft angle, mediolateral offset, and the distance between the lesser trochanter and femoral head center, showed only weak correlations with internal canal geometry. These findings indicate that external femoral morphology alone cannot reliably predict the internal canal characteristics responsible for implant fit, fixation stability, and load transfer, emphasizing the importance of patient-specific three-dimensional morphological assessment during preoperative planning (Veldman et al., 2025).

The clinical relevance of these observations is supported by several independent investigations. Maton et al. reported that proximal femoral morphology, particularly a high cortical index corresponding to the so-called champagne-flute femur, was significantly associated with postoperative radiolucency following cementless total hip arthroplasty, highlighting the influence of cortical architecture on implant osseointegration and long-term stability (Maton et al., 2024). Likewise, Wang et al. demonstrated that iatrogenic comminution during antegrade intramedullary nailing most frequently occurs at the femoral isthmus and is associated with significantly higher rates of fracture nonunion, underscoring the biomechanical importance of this anatomical region during implant insertion (Wang J.-H. et al., 2022). Furthermore, Xu et al. identified advanced age and intramedullary nail instability as independent predictors of femoral shaft fracture nonunion, further supporting the concept that age-related anatomical and mechanical alterations substantially influence fixation success (Xu M. et al., 2026). Together, these studies demonstrate that the femoral canal should not be regarded as a uniform cylindrical structure but rather as a highly heterogeneous anatomical system influenced by age, sex, cortical architecture, and bone quality. Such variability directly affects implant-bone conformity, stress distribution, and fixation stability, reinforcing the need for anatomically adaptive implant designs and advanced functional biomaterials capable of accommodating patient-specific skeletal morphology. Table 1 represents published studies describing age-related femoral remodeling, femoral canal morphology, and their implications for orthopedic implant performance. Collectively, these investigations demonstrate that patient-specific anatomical variation, cortical architecture, and skeletal aging substantially influence implant fit, osseointegration, fixation stability, and fracture healing, supporting the development of personalized intramedullary fixation strategies.

**TABLE 1:** Representative studies investigating femoral morphology, age-related remodeling, and their clinical implications for intramedullary fixation and orthopedic implant performance.

Study	Study design/Population	Imaging or evaluation method	Principal findings	Clinical significance
Veldman et al. (2025)	Retrospective morphometric analysis of 90 very elderly femora (80–105 years)	Three-dimensional computed tomography reconstruction and cross-sectional morphometric analysis	Internal femoral canal morphology showed poor correlation with external proximal femoral anatomy. Significant sex-related differences in cortical thickness and canal geometry were observed	Demonstrates that external anatomical landmarks cannot reliably predict internal canal morphology, supporting patient-specific preoperative planning and implant design
Maton et al. (2024)	Retrospective analysis of 122 patients undergoing cementless total hip arthroplasty	Radiographic assessment using Dorr classification, cortical index, canal flare index, and canal-to-calcar isthmus ratio	A high cortical index (>0.62), characteristic of the “champagne-flute” femur, was significantly associated with postoperative radiolucency	Highlights the influence of proximal femoral morphology on implant osseointegration and long-term fixation stability
Wang J.-H. et al. (2022)	Retrospective study of 211 femoral shaft fractures treated with antegrade intramedullary nailing	Clinical and radiographic evaluation	Iatrogenic comminution occurred in approximately 21% of cases, predominantly at the femoral isthmus, and significantly increased fracture nonunion rates	Identifies the femoral isthmus as a biomechanically vulnerable region during nail insertion and emphasizes the importance of implant–bone compatibility
Xu M. et al. (2026)	Retrospective case-control study of 144 femoral shaft fractures	Multivariate logistic regression and predictive nomogram	Advanced age, intramedullary nail instability, fracture displacement, delayed weight-bearing, and multiple fractures were independent predictors of nonunion	Demonstrates that age-related skeletal changes and mechanical instability substantially influence fracture healing following intramedullary fixation
Rollet (2025)	Review of age-related femoral remodeling	Morphological and biomechanical synthesis	Aging is characterized by progressive endosteal expansion, cortical thinning, deterioration of bone quality, and altered load-bearing characteristics	​

Endosteal expansion represents one of the most consistent manifestations of skeletal aging and is characterized by progressive enlargement of the medullary canal accompanied by cortical thinning and reduced structural support. Together with osteoporosis-related deterioration of bone quality, these changes alter the biomechanical environment surrounding the implant, increase the likelihood of implant-bone mismatch, and contribute to fixation instability and delayed fracture healing (Rollet, 2025; Xu M. et al., 2026). These observations further emphasize that successful intramedullary fixation depends not only on implant design but also on a comprehensive understanding of age-dependent femoral remodeling and patient-specific anatomical variation.

## 3 Current challenges and limitations of conventional intramedullary nail systems

**SCHEME 2:**
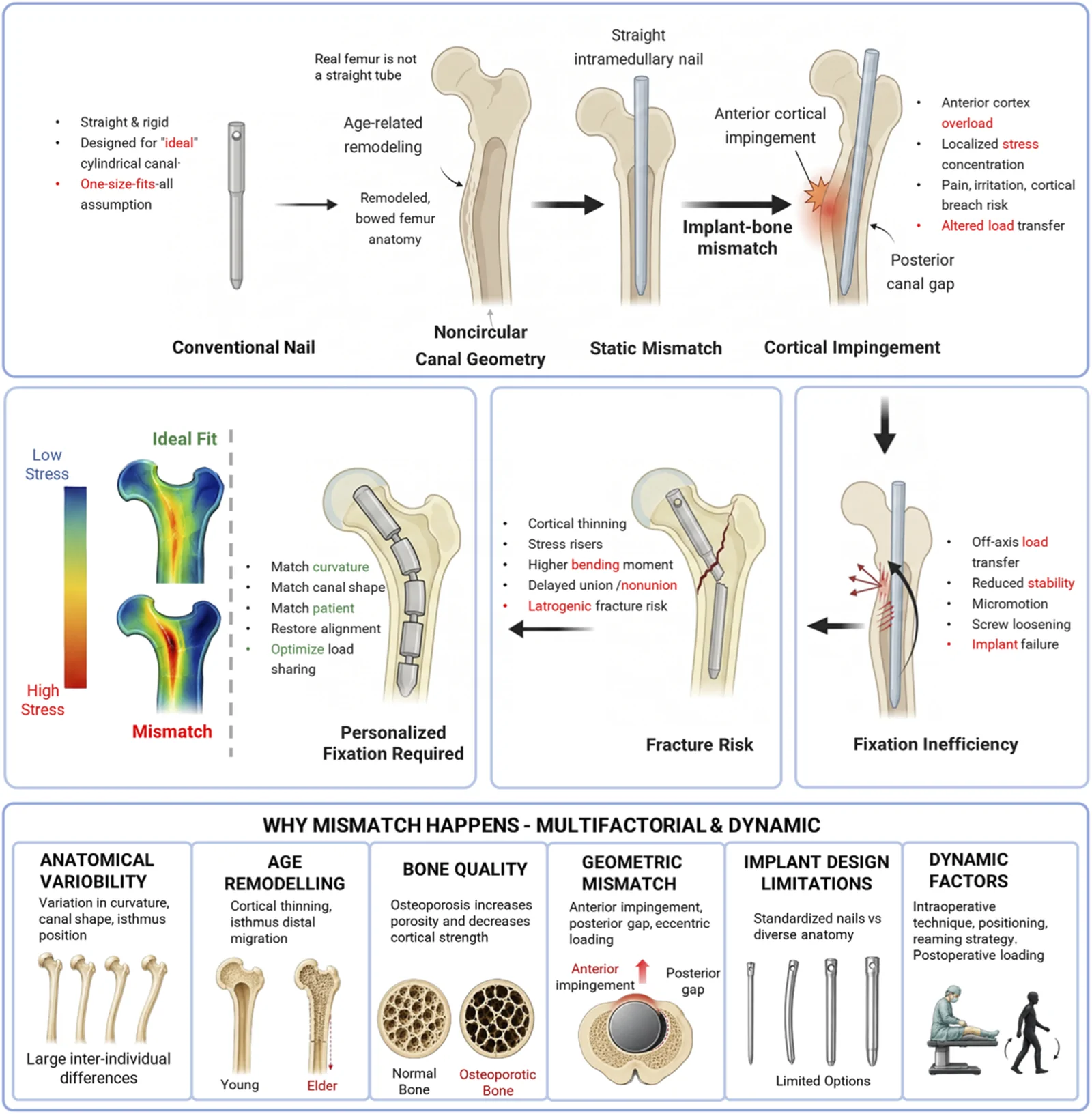
This schematic illustration demonstrates the anatomical and biomechanical constraints of conventional intramedullary nail systems, emphasizing the current problems of conventional nailing systems.

Because of its minimal invasiveness, improved biomechanical stability, and ability to allow for early mobilization, intramedullary nailing remains the gold standard for treating femoral shaft fractures. Postoperative complications, including implant malalignment, anterior cortical impingement, cortical perforation, delayed union, implant failure, and fixation instability, persist despite decades of advancements in implant design and surgical procedures (Čavka and Bašković, 2026). Many of these issues stem from the intrinsic anatomical diversity of the femur, which is occasionally not adequately accommodated by traditional intramedullary nail designs. The femoral isthmus varies substantially in geometry, curvature, and geographic position, as shown in Scheme 2. Canal architecture changes with age. These findings call into question the long-held certainty that a standardized implant can fit a wide range of anatomical structures. As a result, a life-threatening appraisal of present intramedullary fixation systems is compulsory to identify the anatomical and biomechanical parts that lead to implant-related problems, as well as to specify the design criteria for future orthopedic devices. This section examines the major limitations of conventional intramedullary nail systems, with a focus on implant-bone mismatch, age-dependent anatomical changes, and the unmet need for individualized and adaptable fixation techniques.

**SCHEME 3:**
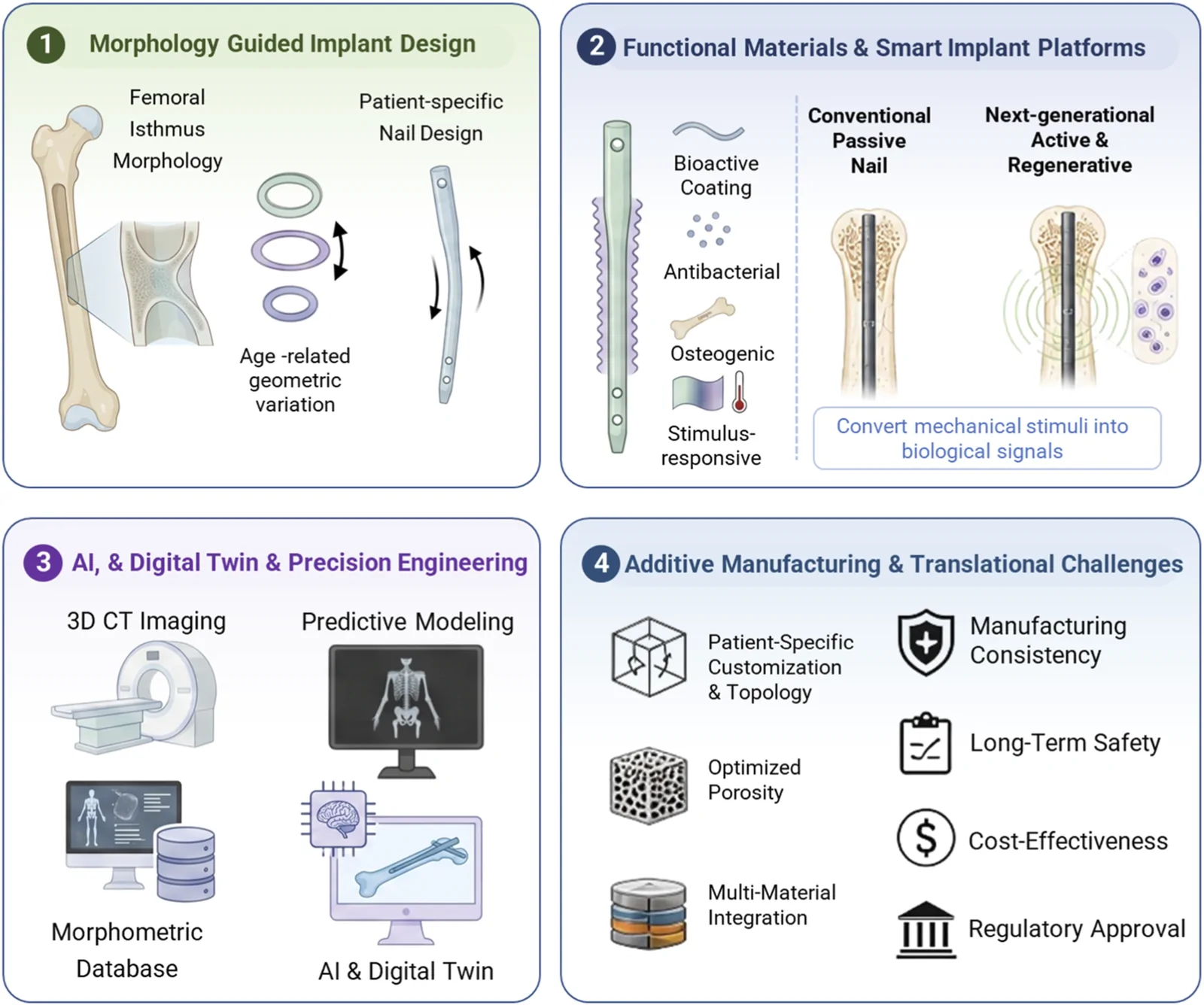
Future roadmap toward personalized and intelligent intramedullary fixation systems integrating morphological-based implants design, artificial intelligence, digital twin modeling, advanced functional materials, and additive manufacturing.

### 3.1 Evolution and design principles of contemporary intramedullary nails

Beyond mechanical fixation, the long-term performance of intramedullary nails is governed by the complex physiological microenvironment established immediately after implantation. Implant placement initiates a cascade of biological events involving protein adsorption, platelet activation, inflammatory cell recruitment, macrophage polarization, angiogenesis, and subsequent osteoblast-mediated bone formation (Uehara et al., 2026). The nature of these early cell-material interactions strongly influences osseointegration, fibrous tissue formation, and long-term implant stability. Conventional metallic implants, despite their excellent mechanical properties, generally function as biologically passive materials and possess limited capacity to actively regulate the local cellular and biochemical environment (Xu B. et al., 2026). Moreover, their relatively high elastic modulus may induce stress shielding and reduce physiological mechanical stimulation required for normal bone remodeling.

To address these limitations, considerable attention has been directed toward surface engineering and functional coatings capable of enhancing biological performance without compromising structural integrity. Representative strategies include hydroxyapatite (Ca_10_(PO_4_)_6_(OH)_2_) and bioactive glass coatings for improving bone-implant bonding, titanium dioxide (TiO_2_) nanotube surfaces for promoting osteoblast adhesion, antibacterial coatings incorporating silver (Ag), copper (Cu), or zinc (Zn) nanoparticles to reduce implant-associated infections, and multifunctional polymeric or ceramic coatings for controlled therapeutic delivery (Chen B. et al., 2026; Yang Z. et al., 2026). More recently, piezoelectric ceramics, particularly barium titanate (BTO), have emerged as promising coating materials because they convert physiological mechanical loading into localized electrical stimulation that mimics the endogenous bioelectrical environment of bone (Gamagedara et al., 2026). This electromechanical effect enhances osteogenic differentiation, promotes osseointegration, modulates inflammatory responses, and accelerates bone regeneration, thereby transforming conventional intramedullary nails from passive fixation devices into biologically active therapeutic platforms. These advances provide the scientific rationale for integrating functional materials into orthopedic implants and establish a conceptual bridge to the following sections that discuss advanced bioactive and stimulus-responsive material strategies.

### 3.2 Implant-bone mismatch arising from femoral anatomical variability

One of the most fundamental limitations of contemporary intramedullary nail systems lies in the mismatch between standardized implant geometry and the highly variable morphology of the human femoral canal (Eveson et al., 2026). Although intramedullary nails are designed to achieve endosteal fixation through canal conformity, their structural configuration is largely based on generalized anatomical assumptions that may not fully capture inter-individual and age-related variations in femoral geometry. Published morphometric studies indicate that the femoral isthmus cannot always be adequately represented as a uniform cylindrical structure and may exhibit an elliptical cross-sectional morphology (Sánchez et al., 2026). This deviation from circularity has important implications for implant fit because most commercially available nails are designed with a predominantly circular cross-section (Elleuch et al., 2026). Consequently, fixation may occur through localized point or line contact rather than uniform surface contact, increasing the potential for stress concentration at the bone-implant interface.

### 3.3 Anterior cortical impingement, cortical perforation, and secondary fracture risks

Anterior cortical impingement represents one of the most clinically significant complications associated with intramedullary nailing of the femur, particularly when implant geometry does not fully correspond to the native anterior femoral bow (Van-Breukelen-García et al., 2026). Published anatomical and biomechanical studies have shown that variations in femoral curvature can influence the alignment between the nail and the medullary canal (Puls et al., 2026). When implant curvature does not adequately match patient-specific anatomy, localized stress concentration may occur at the anterior cortical surface, potentially leading to cortical irritation, remodeling, or, in severe cases, cortical perforation. These risks may be further increased in elderly patients, where cortical thinning and reduced structural resistance make the femur more susceptible to mechanical stress.

### 3.4 Influence of femoral curvature and isthmus position on nail alignment and load transfer

The biomechanical performance of intramedullary nail systems is strongly influenced by the geometric relationship between implant trajectory and the native curvature of the femoral shaft. Previous anatomical and biomechanical investigations have reported substantial inter-individual variability in femoral curvature and in the longitudinal position of the femoral isthmus (Taghipour and Vakili-Tahami, 2026). Such variability can affect implant alignment, load transfer, and the distribution of mechanical stress along the femoral shaft (Liu et al., 2025). When the implant does not adequately conform to these anatomical characteristics, non-uniform contact and altered bending moments may occur, potentially contributing to micro-motion at the fracture site and delayed healing.

### 3.5 Age-related morphological changes as drivers of implant failure

Age-related alterations in femoral morphology are increasingly recognized as important factors influencing intramedullary nail performance (Tara et al., 2026). Published studies have reported progressive changes in cortical thickness, medullary canal configuration, and the spatial distribution of anatomical landmarks with advancing age. In elderly and osteoporotic populations, cortical thinning and endosteal remodeling may alter the mechanical interaction between bone and implant, potentially reducing fixation stability and increasing susceptibility to implant-related complications (Lentrodt, 2026; Wang and Cao, 2026). These observations suggest that implant failure in older patients may be influenced not only by material or surgical factors, but also by age-dependent changes in femoral anatomy. Consequently, patient-specific anatomical assessment should be considered when selecting and designing intramedullary fixation systems.

### 3.6 Challenges of intramedullary fixation in elderly and osteoporotic patients

Intramedullary fixation in elderly and osteoporotic populations presents a distinct set of biomechanical and clinical challenges that extend beyond conventional fracture management considerations (Sain et al., 2026). In these patients, age-related skeletal remodeling is compounded by reduced bone mineral density, compromised cortical integrity, and altered load distribution capacity, collectively creating a mechanically vulnerable environment for intramedullary nail fixation. Osteoporotic bone is characterized by decreased cortical thickness and increased porosity, which significantly reduce its ability to withstand localized stress concentrations induced by implant insertion and postoperative loading (Lee et al., 2026). As a result, even when intramedullary nails are correctly positioned, the reduced structural competence of the femoral cortex may limit effective load transfer between the implant and bone (Zhao X. et al., 2026). This condition increases the likelihood of microfractures, implant subsidence, and progressive loosening under cyclic loading conditions.

Emerging evidence from femoral morphometric studies further highlights these challenges. Recent studies investigating age-associated femoral remodeling have suggested progressive alterations in canal geometry, including changes in cross-sectional shape, cortical distribution, and medullary expansion, which may influence the mechanical environment of intramedullary fixation (Zhang R. y. et al., 2021). In osteoporotic populations, these changes are generally more pronounced due to accelerated endosteal resorption and cortical deterioration, resulting in altered canal geometry and reduced mechanical constraint at the implant-bone interface (Rolvien and Amling, 2022). Consequently, the intrinsic stabilizing effect of the isthmus region may be diminished, reducing the effectiveness of standard intramedullary fixation strategies. Age-associated alterations in femoral canal geometry, including changes in the location and dimensions of the narrowest canal region, may influence the relationship between implant geometry and cortical engagement sites (Parsley et al., 2020). This misalignment may compromise distal fixation stability, particularly in cases where standard nail lengths and locking configurations are applied without consideration of patient-specific anatomical variation.

Furthermore, reduced bone quality in osteoporotic patients exacerbates the risk of iatrogenic complications during nail insertion (Kadoura, 2019). Moreover, compromised bone quality in elderly patients increases the risk of intraoperative complications during nail insertion and reduces the capacity for subsequent biological adaptation and remodeling. The combined effects of altered canal morphology, reduced cortical strength, and impaired regenerative capacity contribute to delayed union and fixation failure in high-risk populations. These challenges highlight the necessity for improved preoperative evaluation, personalized implant strategies, and advanced fixation systems capable of simultaneously addressing mechanical compatibility and biological requirements in aging bone.

### 3.7 Unmet clinical needs for personalized and adaptive intramedullary fixation systems

Despite substantial advancements in intramedullary nail design and surgical techniques, persistent complications continue to highlight fundamental gaps between implant engineering and patient-specific femoral anatomy (Gurzì et al., 2024). The collective findings from the present morphometric analysis, particularly the elliptical configuration of the femoral isthmus, its age-related distal migration, and the progressive reduction in geometric eccentricity, underscore the limitations of standardized fixation systems in accommodating anatomical diversity. Current intramedullary nail systems are predominantly based on averaged anatomical models that assume relatively uniform canal geometry and fixed spatial relationships between proximal landmarks and the isthmus region (Manon et al., 2024). However, as demonstrated in this study, femoral morphology exhibits continuous variability driven by age-related remodeling and inter-individual differences in curvature, canal shape, and longitudinal positioning. These variations directly influence implant fit, alignment, and load transfer efficiency, yet are not adequately addressed in conventional implant design frameworks.

As a result, there remains a critical unmet clinical need for intramedullary fixation systems that can adapt to patient-specific anatomical conditions rather than relying on static geometric assumptions (Junior and Trajanovic, 2022). In particular, the mismatch between circular implant cross sections and elliptical femoral canals, as well as the distal migration of the isthmus with advancing age, necessitates a re-evaluation of how implant geometry is defined and optimized (Wang N. et al., 2025). Without such adaptability, the risk of cortical impingement, suboptimal fixation, and mechanical failure remains inherently elevated, especially in elderly and osteoporotic populations. Furthermore, existing implant systems lack responsiveness to dynamic intraoperative and postoperative conditions, such as variations in bone quality, cortical thickness, and curvature mismatch (Dobrotă et al., 2024). This rigidity limits the ability to achieve optimal biomechanical integration across diverse patient populations. Consequently, there is a growing demand for next-generation intramedullary systems that incorporate patient-specific design principles, adaptive geometry, and multifunctional performance capabilities. Emerging advances in functional materials science, computational modeling, and additive manufacturing offer promising pathways to address these limitations (Sadoghi et al., 2026). However, translating these technologies into clinically viable solutions requires a paradigm shift from standardized implant design toward morphology-guided, biologically adaptive, and mechanically responsive fixation strategies.

Therefore, the unmet clinical needs identified in this section provide a direct foundation for the development of advanced functional material-based approaches, which are discussed in the subsequent section as potential solutions to overcome the intrinsic limitations of conventional intramedullary nail systems.

## 4 Functional material strategies for improving intramedullary nail performance

The limitations of conventional intramedullary nail systems have stimulated growing interest in functional materials that enhance both the biological and mechanical performance of orthopedic implants. By incorporating bioactive, antibacterial, adaptive, and stimulus-responsive functionalities, these advanced materials offer new opportunities to improve osseointegration, reduce implant-associated complications, accelerate fracture healing, and better accommodate patient-specific anatomical variations that are often inadequately addressed by traditional implant designs.

### 4.1 Surface engineering approaches for enhanced osseointegration and bone–implant integration

Successful intramedullary fixation depends not only on mechanical stability but also on effective biological integration at the bone-implant interface (Rosa et al., 2017). Conventional metallic implants often exhibit limited bioactivity, which can delay osseointegration and compromise long-term fixation. Surface engineering strategies have therefore been developed to modify implant surfaces, enhance cellular responses, promote bone formation, and improve implant stability without altering the device’s bulk mechanical properties. Following implantation, the initial interaction between the implant surface and the surrounding biological environment plays a decisive role in determining the long-term success of fixation (Liu et al., 2020). Protein adsorption, cellular adhesion, inflammatory responses, and subsequent bone remodeling are all highly influenced by surface chemistry, topography, roughness, and wettability (Batool et al., 2021). An unfavorable surface environment may lead to inadequate osteoblast attachment, fibrous tissue formation, or insufficient bone ingrowth, ultimately reducing implant stability and increasing the risk of loosening or fixation failure. These challenges become particularly relevant in elderly and osteoporotic patients, where impaired bone regenerative capacity and reduced bone quality already compromise implant anchorage. Therefore, considerable efforts have been directed toward engineering implant surfaces that actively promote osteogenic activity while maintaining mechanical integrity. Modern surface modification approaches aim to create bioactive interfaces capable of accelerating bone formation, strengthening bone-implant integration, and improving long-term clinical outcomes (Dong et al., 2020). Among these approaches, nanotopographical modifications, micro/nano hierarchical architectures, and bioactive ceramic coatings have emerged as some of the most extensively investigated strategies for orthopedic applications.

#### 4.1.1 Nanotopographical surface modification

The biological response to an intramedullary implant is strongly influenced by its surface characteristics. Conventional metallic nail surfaces are generally bioinert and provide limited cues for cellular attachment and tissue integration (Biggs et al., 2010). Nanotopographical surface modification has emerged as an effective strategy to mimic the nanoscale features of the natural extracellular matrix, thereby enhancing interactions between the implant and surrounding bone tissue (Karazisis et al., 2017). By introducing nanopatterns, nanotubes, nanorods, or nanoporous structures onto implant surfaces, researchers have demonstrated improved adhesion, proliferation, and osteogenic differentiation of mesenchymal stem cells and osteoblasts. These nanoscale features can also modulate protein adsorption and cellular signaling pathways involved in bone regeneration. Nanotopographical modifications improve osseointegration without compromising the mechanical strength of the underlying implant. For intramedullary nail applications, enhanced bone-implant integration may contribute to improved fixation stability, particularly in elderly patients with compromised bone quality. However, challenges related to large-scale manufacturing, long-term durability, and surface wear remain important considerations for future clinical translation (Shin et al., 2026).

#### 4.1.2 Surface roughening and micro/nano hierarchical architectures

Beyond nanoscale modifications, surface roughening and the construction of hierarchical micro/nano architectures have emerged as effective strategies to further enhance bone–implant integration (Patil, 2024). Compared with smooth metallic surfaces, roughened topographies significantly increase the available surface area, improving protein adsorption and facilitating stronger initial cell attachment. This enhanced interface promotes osteoblast proliferation and accelerates early-stage osseointegration, which is critical for achieving stable fixation in intramedullary nail systems. Micro-scale roughness provides mechanical interlocking between bone tissue and implant surfaces, while nanoscale features regulate cellular behavior at the molecular level (Wang et al., 2018; Zhang H. et al., 2021). The combination of both scales creates a synergistic effect, where microstructures enhance mechanical stability, and nanostructures guide biological responses. Such hierarchical designs more closely resemble the natural extracellular matrix, thereby improving cellular recognition and signaling. For load-bearing implants such as intramedullary nails, these modifications are particularly important in osteoporotic bone, where reduced bone density limits mechanical anchorage (Carotenuto et al., 2022). Surface roughening can partially compensate for weakened bone-implant contact by increasing frictional resistance and improving interfacial stability. However, maintaining long-term durability of these engineered surfaces under cyclic loading conditions remains a key challenge for clinical translation.

#### 4.1.3 Bioactive ceramic and hydroxyapatite coatings

Bioactive ceramic coatings, particularly hydroxyapatite (HA), have been widely explored to improve the biological performance of metallic intramedullary implants. Hydroxyapatite, being chemically similar to the mineral phase of bone, provides an osteoconductive interface that promotes direct bone bonding rather than fibrous tissue encapsulation (Huang and Yoshimura, 2023). This property significantly enhances early-stage osseointegration and long-term implant stability. When applied as a coating on intramedullary nails, HA facilitates ion exchange at the bone–implant interface, stimulating osteoblast activity and accelerating new bone formation. In addition, calcium and phosphate release from ceramic coatings can further support mineralization processes, improving the quality of newly formed bone around the implant (Liu et al., 2022). Despite these advantages, limitations such as coating delamination under mechanical stress, reduced adhesion strength over time, and variability in degradation behavior restrict widespread clinical use. Therefore, while bioactive ceramic coatings represent a promising strategy for improving implant integration, their long-term mechanical reliability remains a critical consideration for load-bearing applications such as femoral intramedullary fixation.

### 4.2 Antibacterial functional coatings for preventing implant-associated infections

Implant-associated infection remains one of the most serious complications in intramedullary fixation, often leading to delayed healing, repeated surgical interventions, and, in severe cases, implant failure. The risk is particularly significant in long bone surgeries where extensive surgical exposure and metallic foreign bodies create favorable conditions for bacterial adhesion and biofilm formation. Conventional systemic antibiotic administration is often insufficient to prevent localized biofilm development on implant surfaces (Roy et al., 2026; Wang H. et al., 2022) Therefore, antibacterial functional coatings have emerged as a critical strategy to provide localized, long-term antimicrobial protection directly at the bone-implant interface, while simultaneously supporting osseointegration.

Silver-based nanostructures are among the most extensively studied antibacterial coatings for orthopedic implants due to their broad-spectrum antimicrobial activity and strong efficacy at low concentrations. Silver ions exert bactericidal effects through multiple mechanisms, including disruption of bacterial cell membranes, interference with DNA replication, and inhibition of essential enzymatic functions (Dhiman et al., 2019). This multi-targeted action reduces the likelihood of resistance development, making silver particularly attractive for implant-associated infection control. When applied to intramedullary nails, silver coatings can significantly reduce early bacterial adhesion and biofilm formation on the implant surface. This is especially important during the initial postoperative period, where bacterial colonization is most likely to occur (Ahmadabadi et al., 2020). However, despite their strong antibacterial performance, concerns remain regarding potential cytotoxicity at higher ion release rates and long-term biocompatibility with surrounding bone tissue. Therefore, careful control of silver release kinetics is essential to balance antimicrobial efficacy with osteogenic compatibility in clinical applications.

Zinc oxide (ZnO) and copper-based nanomaterials have gained considerable attention as alternative antibacterial coatings for orthopedic implants due to their intrinsic antimicrobial activity and relatively favourable biocompatibility (Tierolf, 2018). These metal oxides generate reactive oxygen species and release metal ions that disrupt bacterial cell membranes, induce oxidative stress, and interfere with essential metabolic pathways, ultimately leading to bacterial cell death. In addition to their antibacterial effects, zinc and copper ions play important roles in bone metabolism (Alhomayani et al., 2025). Zinc is known to support osteoblast proliferation and bone mineralization, while copper contributes to angiogenesis, both of which are beneficial for bone healing and implant integration. This dual functionality makes ZnO and copper-based coatings particularly attractive for intramedullary nail applications, where simultaneous infection control and bone regeneration are required (Pellosi et al., 2025). However, similar to other ion-releasing systems, the therapeutic window is narrow, and excessive ion release may lead to cytotoxic effects on surrounding tissues. Therefore, optimizing coating stability and controlled ion release remains a key challenge for their safe and effective clinical translation.

Graphene and its derivatives, including graphene oxide and reduced graphene oxide, have emerged as promising antibacterial platforms for orthopedic implant coatings due to their unique physicochemical properties (Huang et al., 2023). Their antimicrobial activity is primarily attributed to physical membrane disruption, oxidative stress induction, and the ability to trap and isolate bacterial cells on sharp nanoscale edges. These combined effects reduce bacterial adhesion and inhibit early biofilm formation on implant surfaces. For intramedullary nails, graphene-based coatings offer the additional advantage of high mechanical strength, chemical stability, and large surface area, which can be further functionalized with bioactive molecules or ions (Madni et al., 2026). This multifunctionality allows simultaneous enhancement of antibacterial performance and osteogenic activity, making graphene coatings particularly attractive for complex bone healing environments. However, concerns remain regarding long-term biocompatibility, aggregation behavior, and potential inflammatory responses depending on material form and dosage (Park et al., 2022). Therefore, precise structural control and surface engineering are essential to ensure safe integration of graphene-based systems into load-bearing orthopedic applications.

To overcome the trade-off between infection control and bone regeneration, multifunctional coatings have been developed to simultaneously provide antibacterial protection and promote osteogenic activity. Unlike single-function systems, these coatings integrate bioactive ions, polymers, or nanostructures that can regulate both microbial behavior and cellular responses at the implant interface. In intramedullary nail applications, such coatings are particularly valuable because they address two major causes of fixation failure: biofilm-associated infection and insufficient osseointegration. By incorporating synergistic components such as metal ions, bioactive ceramics, or functional nanomaterials, these systems can inhibit bacterial adhesion while enhancing osteoblast proliferation and differentiation. Despite their promising performance, challenges remain in achieving controlled release kinetics, long-term stability, and consistent functionality under physiological loading conditions. Therefore, further optimization is required to ensure that antibacterial efficacy and osteogenic potential are maintained throughout the entire bone healing process without inducing cytotoxic effects or coating degradation.

### 4.3 Shape adaptive and mechanically responsive materials for anatomical conformity

A fundamental limitation of conventional intramedullary nails is their fixed geometry, which restricts their ability to conform to patient-specific variations in femoral curvature and isthmus morphology (Xu R. et al., 2026). As demonstrated in this study, the femoral isthmus exhibits an elliptical cross-section with age-dependent transformation toward a more circular geometry, alongside distal migration along the femoral shaft. These dynamic anatomical variations cannot be adequately accommodated by static implant designs, creating a strong rationale for shape-adaptive and mechanically responsive materials in intramedullary fixation systems (Xu R. et al., 2026).

Shape memory alloys, particularly nickel-titanium-based systems, offer the ability to undergo controlled deformation and recover predefined shapes in response to physiological conditions such as temperature changes (Mehrpouya et al., 2021). This property enables intramedullary nails to adapt more effectively to the curvature of the femoral canal, thereby improving contact uniformity and reducing stress concentrations at the bone-implant interface. Such adaptability is especially beneficial in anatomically complex or osteoporotic bones, where rigid implants often fail to achieve optimal alignment (Jahadakbar, 2016).

Expandable intramedullary nail technologies further extend this concept by allowing intraoperative or post-insertion expansion to achieve improved canal filling (Booth, 2025). This approach directly addresses the implant-bone mismatch associated with elliptical isthmus geometry by enhancing radial contact and stabilizing fixation within the medullary canal. However, precise control over expansion magnitude and long-term mechanical stability remains a key design challenge. Self-adjusting implant architectures represent an emerging direction aimed at enabling continuous adaptation to variable canal geometries. By integrating compliant structural elements or modular configurations, these systems can dynamically respond to local anatomical constraints, potentially reducing the risk of cortical impingement and improving load distribution (Allen, 2025). Shape-adaptive and mechanically responsive materials provide a promising pathway to overcome the rigid design limitations of conventional intramedullary nails. Their ability to conform to patient-specific anatomy directly aligns with the morphological variability identified in this study, offering a functional bridge between anatomical insight and implant design innovation.

### 4.4 Bioactive materials for accelerating bone regeneration and fracture healing

Beyond mechanical stability and geometric conformity, successful intramedullary fixation requires effective biological support for bone regeneration at the fracture site. Bioactive materials have therefore been widely explored to enhance osteogenesis, accelerate mineralization, and improve the quality of newly formed bone (Sun et al., 2025). These materials act by releasing biologically active ions or by providing signaling cues that regulate cellular differentiation and tissue remodeling processes. In the context of intramedullary nails, bioactive modifications are particularly important because the implant is in direct contact with the endosteal surface, where bone remodeling activity is highly dynamic (Rosa, 2016; Sun et al., 2025). Enhancing this interface can improve secondary bone healing, reduce healing time, and increase long-term fixation strength. Moreover, in elderly and osteoporotic patients, where regenerative capacity is diminished, bioactive materials can partially compensate for impaired biological responses. Common bioactive systems include ion-releasing materials and inorganic compounds that stimulate osteoblastic activity and support extracellular matrix formation. These strategies are often integrated with structural or surface modifications to achieve a synergistic effect between mechanical fixation and biological regeneration, ultimately improving overall fracture healing outcomes.

#### 4.4.1 Bioactive glasses

Bioactive glasses are widely recognized for their ability to bond directly with bone through the formation of a hydroxycarbonate apatite layer upon implantation. This surface layer closely resembles the mineral phase of natural bone, enabling strong chemical integration between the implant and surrounding tissue (Kaur et al., 2014). In intramedullary fixation systems, bioactive glass coatings or composites can significantly enhance osteoconduction and promote faster bone regeneration at the fracture interface. The release of biologically active ions such as silicon, calcium, and phosphorus from bioactive glasses further stimulates osteoblast proliferation and differentiation, thereby accelerating bone matrix formation. These ionic interactions also contribute to angiogenesis and improved local metabolic activity, which are essential for effective fracture healing, particularly in compromised bone environments (Kajander et al., 2023). However, the relatively brittle nature of bioactive glasses and their limited mechanical durability restrict their direct application as structural components in load-bearing implants. Therefore, they are primarily utilized as coatings or composite additives in intramedullary nail systems, where they can enhance biological performance without compromising mechanical integrity.

#### 4.4.2 Magnesium-based biomaterials

Magnesium-based biomaterials have attracted significant attention in orthopedic applications due to their constructive mechanical properties, biodegradability, and strong biological activity (Wu et al., 2024). Magnesium is a natural element in bone tissue and plays an essential role in enzymatic processes, cellular metabolism, and bone mineralization. In intramedullary fixation systems, magnesium and its alloys can promote bone regeneration while gradually degrading *in vivo*, thereby reducing long-term implant-related complications. The controlled degradation of magnesium releases Mg^2+^ ions, which stimulate osteoblast proliferation, enhance angiogenesis, and support new bone formation (Xu L.-H. et al., 2025). This biological activity is particularly beneficial in fracture healing environments where rapid tissue regeneration is required. Additionally, the mechanical properties of magnesium alloys are closer to those of natural bone compared to conventional metallic implants, which helps reduce stress shielding effects (Abraham and Subramani, 2023). However, the rapid corrosion rate of magnesium in physiological environments remains a major limitation, often leading to premature loss of mechanical integrity and gas formation. Therefore, surface modification and alloying strategies are commonly employed to control degradation behavior and improve the clinical applicability of magnesium-based systems in load-bearing intramedullary nail applications.

#### 4.4.3 Strontium functionalized coatings

Strontium functionalized coatings have emerged as effective bioactive strategies for enhancing bone regeneration and improving implant integration. Strontium plays a dual role in bone metabolism by stimulating osteoblast-mediated bone formation while simultaneously inhibiting osteoclast-driven bone resorption (Liu et al., 2023). This balanced regulation of bone remodeling makes it particularly valuable for improving fracture healing outcomes in compromised bone conditions. When applied to intramedullary nails, strontium-releasing coatings can enhance local osteogenic activity at the bone-implant interface, leading to improved callus formation and faster mineralization (Akobundu et al., 2025). This effect is especially beneficial in elderly and osteoporotic patients, where reduced bone formation and increased resorption often impair healing efficiency. Despite these advantages, controlling the release rate of strontium ions remains a critical challenge. Excessive or uncontrolled release may disrupt normal bone remodeling balance or reduce long-term coating stability. Therefore, optimization of coating composition and degradation kinetics is essential for achieving safe and sustained therapeutic effects in clinical applications.

#### 4.4.4 Calcium phosphate based regenerative interfaces

Calcium phosphate-based materials represent one of the most widely used bioactive systems for promoting bone regeneration due to their chemical similarity to the mineral component of bone (Hou et al., 2022). These materials, including tricalcium phosphate and related derivatives, support direct bone bonding and provide a favorable microenvironment for osteoblast attachment, proliferation, and extracellular matrix deposition. When integrated into intramedullary nail systems as coatings or interfacial layers, calcium phosphate-based materials enhance osteoconductivity and accelerate the formation of new bone at the implant surface (Van Rijt et al., 2022). Their gradual resorption behavior allows progressive replacement by native bone tissue, contributing to stable long-term fixation without introducing foreign material persistence. In addition, calcium and phosphate ion release plays an important role in regulating local mineralization processes and promoting early-stage callus formation. This is particularly relevant in compromised bone conditions where natural regenerative capacity is reduced (Xu et al., 2023). However, limitations such as relatively low mechanical strength and variable degradation rates restrict their use as standalone structural materials. Therefore, calcium phosphate systems are primarily employed as functional coatings or composite components in intramedullary implants to improve biological performance while maintaining mechanical integrity.

### 4.5 Stimulus responsive functional materials for smart orthopedic implants

Stimulus-responsive functional materials represent the next evolution in intramedullary fixation systems by enabling implants to actively respond to biological, mechanical, and external physical cues (Teixeira do Nascimento et al., 2025). Unlike conventional static implants, these systems can adapt their properties in response to stimuli such as light, ultrasound, mechanical stress, or electrical signals, thereby integrating therapeutic functions directly into the fixation device (Yang S. et al., 2024). In the context of femoral fractures, such materials offer the potential to simultaneously enhance bone regeneration, prevent infection, and improve implant integration under patient-specific physiological conditions.

#### 4.5.1 Light responsive materials for antibacterial and osteogenic orthopedic applications

Light-responsive materials offer a non-invasive and precisely controllable approach to activate therapeutic functions within orthopedic implants. Upon exposure to external light sources, particularly in the near-infrared region, these materials can generate localized photothermal effects or reactive oxygen species that enable antibacterial action while simultaneously influencing cellular behavior (Liao et al., 2025). In intramedullary nail applications, light-responsive coatings can be designed to provide on-demand antimicrobial activity during the critical postoperative period when infection risk is highest. In addition, controlled light stimulation can enhance osteogenic signaling pathways, thereby supporting bone regeneration at the implant interface (Villegas et al., 2024). The ability to spatially and temporally regulate these responses provides a significant advantage over conventional passive antibacterial systems. However, limitations such as light penetration depth in deep tissues and potential thermal damage to surrounding healthy bone must be carefully considered. Therefore, optimization of light wavelength, intensity, and material sensitivity is essential for safe and effective clinical translation in load-bearing orthopedic systems.

#### 4.5.2 Piezoelectric biomaterials for electromechanically active orthopedic fixation

Bone is not merely a mechanically supporting tissue but also an inherently electromechanically active biological system. The piezoelectric nature of bone originates primarily from the deformation of collagen fibrils, which generate localized electrical potentials under mechanical loading (Wu et al., 2025). These endogenous electrical signals participate in regulating bone remodeling by influencing osteoblast differentiation, extracellular matrix formation, calcium deposition, and mineralization processes (Yao et al., 2025). Therefore, recreating the bioelectrical microenvironment of native bone through artificial piezoelectric materials has emerged as an attractive strategy for enhancing bone regeneration and improving implant integration.

The fundamental mechanism of piezoelectric bone regeneration relies on the conversion of mechanical stimulation into localized electrical signals (Mondal et al., 2025; Zhang et al., 2024). Under physiological loading or externally applied ultrasound stimulation, piezoelectric materials undergo deformation-induced polarization, resulting in charge separation and the generation of surface electrical potentials. These electrical cues can regulate cellular behaviors through multiple biological pathways, including enhanced intracellular Ca^2+^ signaling, activation of bone morphogenetic protein-2 (BMP-2) pathways, upregulation of runt-related transcription factor 2 (RUNX2), increased alkaline phosphatase (ALP) activity, and enhanced osteocalcin expression (Holghoomi et al., 2026; Lee et al., 2017). Consequently, piezoelectric stimulation promotes osteogenic differentiation, extracellular matrix maturation, and accelerated bone–implant integration.

Among piezoelectric ceramics, barium titanate has attracted extensive attention due to its high dielectric constant, stable piezoelectric response, chemical stability, and excellent biocompatibility (Rakesh et al., 2026). As a lead-free piezoelectric ceramic, BTO provides strong electromechanical coupling while avoiding the toxicity concerns associated with lead-based materials (Ibrahim, 2024). Similarly, potassium sodium niobate (K_0.5_Na_0.5_NbO_3_, KNN) has emerged as a promising lead-free alternative because of its high piezoelectric coefficient and favorable biological compatibility (Yang S. et al., 2026). In addition, zinc oxide (ZnO) nanostructures offer nanoscale piezoelectric stimulation together with intrinsic antibacterial properties, making them attractive for multifunctional orthopedic applications (Weng et al., 2022).

Beyond ceramic systems, piezoelectric polymers have been explored to overcome the brittleness and limited flexibility of inorganic materials. Poly (vinylidene fluoride) (PVDF) exhibits strong piezoelectricity due to its electroactive β-phase, excellent flexibility, and mechanical compatibility with soft biological environments (Zhao T. et al., 2026). However, its limited biodegradability and relatively weak biological activity restrict its direct application in regenerative orthopedic systems. In contrast, poly (L-lactic acid) (PLLA) possesses intrinsic piezoelectric characteristics after mechanical stretching or electrical polarization and offers advantages including biodegradability and favorable biocompatibility (Babichuk et al., 2022).

**FIGURE 3:**
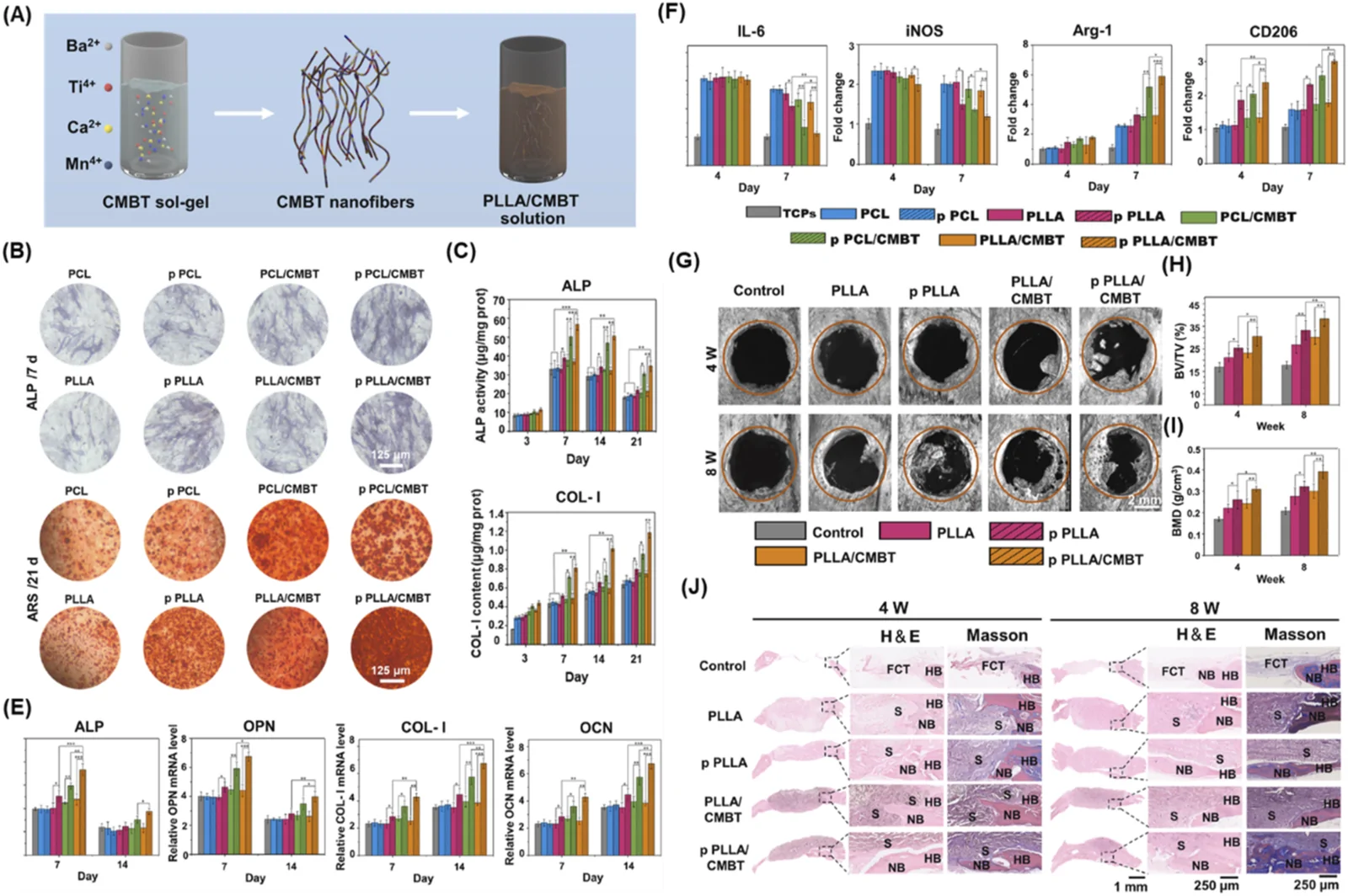
Multifunctional osteogenic and immunomodulatory performance of polarized PLLA/CMBT piezoelectric scaffolds. **(A)** Schematic illustration of the fabrication process of Ca/Mn co-doped BaTiO_3_ (CMBT) nanofibers incorporated into a biodegradable poly (L-lactide) (PLLA) matrix, followed by electrical polarization to construct an electroactive and bioactive scaffold. **(B–F)** *In vitro* evaluation of osteogenic differentiation of bone marrow mesenchymal stem cells (BMSCs) and macrophage polarization of RAW264.7 cells cultured on polarized and unpolarized membranes. **(B)** Alkaline phosphatase (ALP) staining and Alizarin Red staining for osteogenic mineralization; **(C)** quantitative analysis of ALP activity; **(D)** collagen-I (COL-I) production; **(E)** expression levels of osteogenic genes, including ALP, osteopontin (OPN), COL-I, and osteocalcin (OCN); **(F)** macrophage phenotype-related gene expression of pro-inflammatory M1 markers (IL-6 and iNOS) and anti-inflammatory M2 markers (CD206 and Arg-1). **(G–J)** *In vivo* assessment of bone regeneration using rat calvarial defect models implanted with polarized or unpolarized PLLA/CMBT scaffolds. **(G)** Three-dimensional reconstructed micro-computed tomography (micro-CT) images of regenerated bone; quantitative evaluation of new bone formation based on **(H)** bone volume fraction (BV/TV) and **(I)** bone mineral density (BMD); **(J)** Histological analysis using hematoxylin and eosin (H&E) and Masson’s trichrome staining. S, residual scaffold; FCT, fibrous connective tissue; HB, host bone; NB, newly formed bone. Statistical significance: *p* < 0.05, p < 0.01, and *p* < 0.001 (n = 4). Adapted with permission from Composites Part B: Engineering. Copyright ©, Elsevier (Zheng et al., 2022).

To further combine mechanical adaptability, biological activity, and electromechanical functionality, piezoelectric nanocomposites have been developed by integrating ceramic nanoparticles with biodegradable polymer matrices. Representative systems include BTO/PLLA, BTO/PCL, BTO/chitosan, and graphene-based piezoelectric composites. These hybrid platforms provide enhanced mechanical strength, improved electrical stimulation efficiency, and optimized cellular interactions compared with individual components. For example, Zheng et al. developed Ca/Mn co-doped BTO (CMBT) nanofibers incorporated into a biodegradable PLLA matrix to construct multifunctional piezoelectric scaffolds (Figure 3) (Zheng et al., 2022). The incorporation of osteogenic Ca and Mn ions improved biological activity while maintaining the intrinsic piezoelectric characteristics of BTO. After polarization, PLLA/CMBT composites generated enhanced electromechanical stimulation, significantly promoting bone marrow mesenchymal stem cell (BMSC) proliferation and osteogenic differentiation. Furthermore, these composites exhibited antibacterial and anti-inflammatory properties and accelerated bone regeneration in a rat calvarial defect model, demonstrating the potential of piezoelectric biomaterials as multifunctional regenerative platforms (Zheng et al., 2022).

For intramedullary fixation, ultrasound-responsive piezoelectric materials provide additional opportunities because ultrasound can penetrate deep tissues and activate electromechanical stimulation without invasive electrical connections (Nizami et al., 2026; Zhang S. et al., 2026). Such systems enable controllable stimulation intensity and timing, offering potential for personalized fracture-healing strategies. However, several challenges remain before clinical translation, including insufficient electrical output under physiological mechanical loading, long-term piezoelectric stability, integration with metallic implant systems, scalable manufacturing, biosafety evaluation, and clinical validation. Addressing these challenges through material optimization, surface engineering, and intelligent implant design will be essential for transforming piezoelectric biomaterials from experimental platforms into next-generation regenerative orthopedic technologies (Roy J. et al., 2025; Zheng et al., 2022).

#### 4.5.3 Triboelectric systems for self-powered orthopedic monitoring and regeneration

Triboelectric systems have emerged as an innovative class of self-powered functional materials that convert mechanical motion into electrical signals through contact electrification and electrostatic induction (Roy S. et al., 2025). In orthopedic applications, these systems can harvest biomechanical energy generated during daily activities such as walking or weight bearing, enabling continuous, self-sustained electrical stimulation without external power sources. For intramedullary fixation, triboelectric materials offer dual functionality: energy harvesting and biological stimulation (Iqbal et al., 2024; Su et al., 2025). The generated electrical output can promote osteogenic differentiation and enhance bone regeneration by providing microelectrical cues similar to endogenous bioelectric signals involved in bone remodeling. At the same time, these systems can be integrated into implants for real-time monitoring of mechanical strain and healing progression (Silva et al., 2026). Despite these advantages, challenges remain in achieving stable long-term output under physiological conditions and ensuring reliable integration within metallic implant systems. Material fatigue, signal degradation, and interface stability are key barriers that must be addressed before clinical translation in load-bearing environments.

Although piezoelectric and triboelectric biomaterials have demonstrated remarkable potential for promoting osteogenesis, antibacterial activity, and tissue regeneration, the current evidence base remains largely limited to *in vitro* investigations and small-animal models (Zhang Z. et al., 2026). This limitation reflects the emerging nature of these technologies in regenerative orthopedics, where early studies have primarily focused on establishing fundamental electromechanical mechanisms and biological responses rather than immediate clinical implementation. In particular, triboelectric systems require further evaluation under realistic orthopedic conditions involving repeated mechanical loading, physiological fluid exposure, long-term implantation, and complex bone–implant interactions. Future studies should prioritize standardized evaluation protocols, large-animal validation, mechanical durability assessment, and clinical feasibility studies to overcome the translational gap between laboratory demonstrations and high-load applications such as femoral intramedullary fixation.

#### 4.5.4 Biomimetic and stimuli responsive platforms for treating implant associated bone infections

Biomimetic and stimuli-responsive platforms have been developed to address implant-associated infections by integrating infection sensing and on-demand therapeutic release within a single system (Dini et al., 2025). These smart platforms are designed to respond to infection-related microenvironmental changes such as pH reduction, elevated enzyme levels, or inflammatory signals, thereby enabling localized and timely antimicrobial action. In intramedullary nail applications, such systems offer a significant advantage over conventional antibiotic delivery approaches by minimizing systemic exposure while maximizing local efficacy at the bone-implant interface. Upon detection of infection triggers, these materials can release antibacterial agents, generate reactive oxygen species, or activate photothermal or electrochemical responses to disrupt bacterial biofilms (Dini et al., 2025). The biomimetic aspect of these platforms lies in their ability to mimic natural immune responses, providing adaptive and self-regulating defense mechanisms. However, challenges including precise trigger specificity, controlled release kinetics, and long-term stability in complex physiological environments remain critical barriers for clinical translation.

The transition from conventional intramedullary fixation toward next-generation orthopedic systems requires a comprehensive understanding of how different functional material strategies address specific clinical challenges associated with implant performance. Although various approaches, including bioactive coatings, antibacterial nanomaterials, piezoelectric platforms, shape-adaptive materials, and artificial intelligence-assisted design systems, have demonstrated significant potential, each strategy provides distinct biological and mechanical advantages while facing specific translational limitations (Gnanakani and Vijayalakshmi, 2024; Sharma et al., 2026). Bioactive coatings primarily enhance bone-implant integration through osteoconductive interactions, whereas antibacterial nanomaterials aim to mitigate implant-associated infections through controlled antimicrobial effects (Kazimierczak and Przekora, 2020). In contrast, piezoelectric materials introduce an active therapeutic dimension by converting mechanical stimuli into localized electrical signals to regulate osteogenesis and accelerate bone regeneration. Shape-adaptive materials offer improved anatomical conformity through dynamic structural responses, while AI-driven computational approaches enable personalized implant optimization based on patient-specific anatomical and biomechanical information (Kumar et al., 2024). A comparative overview of these emerging strategies, highlighting their representative materials, mechanisms, advantages, limitations, and current translational status, is summarized in Table 2. Such integration of material functionality with personalized design principles provides a roadmap for developing intelligent intramedullary fixation systems capable of simultaneously addressing mechanical compatibility, biological regeneration, and patient-specific clinical requirements.

**TABLE 2:** Comparison of functional material strategies for next-generation intramedullary fixation systems.

Material strategy	Representative materials	Primary mechanism	Key advantages	Major limitations	Translational stage
Bioactive coatings	Hydroxyapatite, bioactive glass	Ion release and osteoconductive interface formation	Enhanced osseointegration	Limited mechanical contribution	Clinical application
Antibacterial nanomaterials	Ag, Cu, Zn nanoparticles	Antimicrobial ion-mediated effects	Reduced implant infection	Potential cytotoxicity	Preclinical/early clinical
Piezoelectric materials	BaTiO_3_, ZnO, PVDF	Mechanical-to-electrical conversion	Osteogenic stimulation and smart response	Long-term stability and optimization required	Preclinical
Shape-adaptive materials	Shape-memory alloys, responsive polymers	Dynamic structural adaptation	Improved anatomical conformity	Manufacturing complexity	Experimental
AI/digital twin systems	AI + computational modeling	Predictive personalized design	Precision planning and optimization	Data and regulatory challenges	Emerging

## 5 Future perspectives and emerging research opportunities

The limitations identified in conventional intramedullary nail systems, together with advances in functional materials and computational modeling, collectively indicate a clear shift toward next-generation orthopedic implants that are patient-specific, adaptive, and multifunctional. Future development is expected to move beyond static fixation concepts toward dynamic systems that integrate structural adaptation, biological responsiveness, and digital intelligence. In this context, femoral morphology, particularly the elliptical nature of the isthmus and its age-dependent variation, should be considered a central design parameter rather than a secondary anatomical reference.

### 5.1 Personalized and morphology-guided implant design

Future intramedullary fixation strategies are expected to move beyond universal implant configurations toward patient-specific designs generated from individualized anatomical information. Advanced imaging, particularly three-dimensional computed tomography reconstruction, provides opportunities to quantify femoral geometry and establish population-specific anatomical databases (Asano et al., 2025). These datasets can guide the optimization of nail diameter, curvature, locking position, and implant–bone contact according to patient-specific characteristics. Such approaches may significantly reduce anatomical mismatch and improve fixation reliability, particularly in elderly and osteoporotic populations where morphological variation and compromised bone quality present additional challenges (Wang Y. et al., 2025).

### 5.2 Integration of functional materials and smart implant platforms

The future of orthopedic fixation will depend not only on improved implant geometry but also on the incorporation of multifunctional materials capable of interacting with the biological environment (Zhang et al., 2024). Bioactive coatings, antibacterial nanostructures, osteogenic interfaces, and stimulus-responsive platforms provide opportunities to enhance osseointegration, reduce infection risks, and accelerate bone regeneration. Among these emerging approaches, piezoelectric biomaterials represent a particularly promising direction because they enable implants to convert mechanical stimuli generated during daily activities into localized electrical signals that regulate cellular responses (Kutner et al., 2021). The combination of morphology-guided design with piezoelectric and other functional material platforms may transform intramedullary nails from passive stabilizing devices into active regenerative systems.

### 5.3 Artificial intelligence, digital twins, and precision orthopedic engineering

Artificial intelligence and digital twin technologies are expected to become critical components of future implant development and surgical planning. By integrating medical imaging data, biomechanical simulations, and clinical outcomes, AI-driven platforms may enable prediction of implant compatibility, fracture healing trajectories, and potential failure risks before surgery (Dean et al., 2024). Digital twin models further provide a virtual environment for evaluating implant performance under patient-specific loading conditions, allowing iterative optimization of implant design and surgical strategies (Al-Smadi et al., 2026). However, challenges related to data standardization, computational accuracy, regulatory approval, and clinical validation must be addressed before widespread implementation.

### 5.4 Additive manufacturing and translational challenges

The combination of advanced manufacturing technologies with functional biomaterials offers new opportunities for producing anatomically customized and multifunctional implants. Additive manufacturing enables precise control over implant architecture, porosity, surface topology, and material distribution, facilitating improved mechanical compatibility and biological integration (Warke et al., 2026). Nevertheless, successful clinical translation requires overcoming challenges related to manufacturing reproducibility, long-term safety evaluation, cost-effectiveness, and regulatory acceptance.

### 5.5 Roadmap toward next-generation intramedullary fixation systems

The development of next-generation intramedullary fixation systems requires a coordinated roadmap that integrates anatomical insight, material innovation, and digital healthcare technologies (Luo, 2024). Based on current evidence, including femoral isthmus morphology, age-related geometric transformation, and implant–bone mismatch, future systems should prioritize adaptability over standardization. The first step involves improving anatomical data acquisition and interpretation through high-resolution imaging and population-specific morphometric databases. This will allow more accurate characterization of femoral variability, particularly in terms of isthmus shape, curvature, and spatial positioning (Siewerdsen, 2025). The second step focuses on translating this anatomical knowledge into design frameworks that incorporate patient-specific customization of implant geometry and mechanical behavior. Parallel to design evolution, functional materials must be further optimized to provide integrated biological and mechanical functions, including antibacterial activity, osteogenic stimulation, and stimulus-responsive adaptability (Li et al., 2026). Finally, digital tools such as artificial intelligence and digital twin modeling should be embedded into preoperative planning and implant selection to ensure predictive, precise, and reproducible outcomes. Jointly, this roadmap highlights a transition from conventional one-size-fits-all implants toward intelligent, adaptive, and biologically active fixation systems tailored to individual patient anatomy and physiological needs.

## 6 Conclusion

Femoral intramedullary fixation remains a fundamental strategy for the management of femoral fractures; however, increasing clinical evidence demonstrates that anatomical diversity, age-associated skeletal remodeling, and the limited biological functionality of conventional implants continue to restrict long-term therapeutic outcomes. Variations in femoral canal geometry, isthmus morphology, cortical architecture, femoral curvature, and bone quality substantially influence implant-bone compatibility, stress distribution, fixation stability, and fracture healing, highlighting the limitations of generalized implant designs based on simplified anatomical assumptions. These challenges are particularly pronounced in elderly and osteoporotic populations, where progressive endosteal expansion, cortical thinning, and compromised regenerative capacity further increase the risk of fixation failure and delayed healing.

To overcome these limitations, recent advances in materials chemistry and biomedical engineering have enabled the development of multifunctional orthopedic strategies, including bioactive surface modifications, antibacterial nanostructured coatings, osteogenic interfaces, shape-adaptive materials, and stimulus-responsive implant platforms. Among these emerging approaches, piezoelectric biomaterials represent a particularly promising direction due to their unique ability to transform mechanical stimuli generated during physiological activities into localized electrical signals that resemble the endogenous electromechanical environment of bone. By regulating cellular behaviors, promoting osteogenic differentiation, enhancing osseointegration, modulating inflammatory responses, and accelerating bone regeneration, piezoelectric systems provide new opportunities to transform traditional metallic implants from passive mechanical stabilizers into biologically active regenerative platforms.

Future orthopedic fixation systems are expected to emerge from the integration of advanced functional materials with patient-specific imaging, three-dimensional anatomical reconstruction, artificial intelligence-assisted design, digital twin modeling, additive manufacturing, and precision surgical technologies. Such interdisciplinary convergence will enable the development of adaptive implants capable of simultaneously addressing anatomical variability, mechanical requirements, and biological healing processes. Ultimately, the integration of anatomical intelligence with multifunctional nanomaterials and stimulus-responsive piezoelectric platforms offers a transformative pathway toward next-generation personalized intramedullary fixation systems, redefining orthopedic implants as intelligent regenerative devices rather than conventional mechanical supports and providing new solutions for the growing challenges of an aging global population.
